# Application of *in silico* bulked segregant analysis for rapid development of markers linked to *Bean common mosaic virus* resistance in common bean

**DOI:** 10.1186/1471-2164-15-903

**Published:** 2014-10-16

**Authors:** Marco H Bello, Samira M Moghaddam, Mark Massoudi, Phillip E McClean, Perry B Cregan, Phillip N Miklas

**Affiliations:** Vegetable and Forage Crops Research Unit, USDA, Agricultural Research Service, Prosser, WA 99350 USA; Department of Plant Sciences and Genomics and Bioinformatics Program, North Dakota State University, Fargo, ND 58108 USA; Ag-Biotech Inc, 2191 San Juan Hollister Rd, San Juan Bautista, CA 95045 USA; Soybean Genomics and Improvement Laboratory, USDA, Agricultural Research Service, Beltsville, MD 20705 USA

**Keywords:** Marker-assisted selection, Molecular breeding, KASP, CAPS, Disease resistance

## Abstract

**Background:**

Common bean was one of the first crops that benefited from the development and utilization of molecular marker-assisted selection (MAS) for major disease resistance genes. Efficiency of MAS for breeding common bean is still hampered, however, due to the dominance, linkage phase, and loose linkage of previously developed markers. Here we applied *in silico* bulked segregant analysis (BSA) to the BeanCAP diversity panel, composed of over 500 lines and genotyped with the BARCBEAN_3 6K SNP BeadChip, to develop codominant and tightly linked markers to the *I* gene controlling resistance to *Bean common mosaic virus* (BCMV).

**Results:**

We physically mapped the genomic region underlying the *I* gene. This locus, in the distal arm of chromosome Pv02, contains seven putative NBS-LRR-type disease resistance genes. Two contrasting bulks, containing BCMV host differentials and ten BeanCAP lines with known disease reaction to BCMV, were subjected to *in silico* BSA for targeting the *I* gene and flanking sequences. Two distinct haplotypes, containing a cluster of six single nucleotide polymorphisms (SNP), were associated with resistance or susceptibility to BCMV. One-hundred and twenty-two lines, including 115 of the BeanCAP panel, were screened for BCMV resistance in the greenhouse, and all of the resistant or susceptible plants displayed distinct SNP haplotypes as those found in the two bulks. The resistant/susceptible haplotypes were validated in 98 recombinant inbred lines segregating for BCMV resistance. The closest SNP (~25-32 kb) to the distal NBS-LRR gene model for the *I* gene locus was targeted for conversion to codominant KASP (Kompetitive Allele Specific PCR) and CAPS (Cleaved Amplified Polymorphic Sequence) markers. Both marker systems accurately predicted the disease reaction to BCMV conferred by the *I* gene in all screened lines of this study.

**Conclusions:**

We demonstrated the utility of the *in silico* BSA approach using genetically diverse germplasm, genotyped with a high-density SNP chip array, to discover SNP variation at a specific targeted genomic region. In common bean, many disease resistance genes are mapped and their physical genomic position can now be determined, thus the application of this approach will facilitate further development of codominant and tightly linked markers for use in MAS.

**Electronic supplementary material:**

The online version of this article (doi:10.1186/1471-2164-15-903) contains supplementary material, which is available to authorized users.

## Background

Traditional plant breeding relies on the discovery, phenotypic selection, and introgression of desirable traits to develop superior cultivars (e.g., with improved agronomic traits, pest and disease resistance, abiotic stress tolerance). This process usually takes 7-10 years and significant economic resources [1]. However, the application of marker-assisted selection (MAS), for the detection of genes or genomic regions underlying a trait of interest, can increase the genetic gain over phenotypic selection in breeding programs by reducing time and costs [2, 3].

In the past three decades, molecular markers have been employed in common bean (*Phaseolus vulgaris* L.) for studies of genetic diversity, germplasm characterization, genetic mapping of major genes or quantitative trait loci (QTL) controlling agronomic traits or tolerance to abiotic stresses, and tagging and MAS of disease resistance genes [4–6]. Early discovery of molecular markers [e.g., restriction fragment length polymorphism (RFLP), random amplified polymorphic DNA (RAPD) sequence, and amplified length polymorphism (AFLP)] often focused on variation between genetically distant materials from different market classes belonging to the Middle American and Andean gene pools [4]. In the past, genetic maps were mostly generated from wide crosses and were not sufficiently dense with markers. In addition, markers linked to a given trait were often times not found in other unrelated populations, restricting their potential use for MAS [5] to certain populations or gene pools in common bean.

As an alternative to bi-parental linkage mapping for the identification of markers tightly linked to monogenic and quantitative disease resistance loci, bean geneticists developed molecular tagging methods utilizing several types of segregating populations, including F_2_, near-isogenic lines (NILs), backcrossed inbred lines (BIL), and recombinant inbred lines (RILs) [7–9], often in combination with bulked segregant analysis (BSA) [10–12]. BSA was originally developed to identify markers linked to disease resistance genes in plants, without the need of a genetic map, and consisted of genotyping two bulks, each containing individual plants with extreme phenotypes (i.e., resistant or susceptible). Polymorphic markers detected between the bulks are often located in genomic regions linked to the trait, and then candidate markers are screened across the original segregating population and additional lines and populations to confirm the marker-trait linkage [10]. These markers were also used, together with already available framework maps, to estimate the genomic position of disease resistance loci onto the consensus genetic map of common bean [5, 6, 13].

Since the tagging of the *Ur-4* bean rust (*Uromyces appendiculatus*) gene [11] many markers linked to genes and QTL controlling resistance to fungal, bacterial, and viral pathogens were developed by BSA approach using RAPD markers [5]. The conversion of RAPD to SCAR (sequence characterized amplified region) markers facilitated MAS (e.g., indirect phenotypic selection, selection of hypostatic genes, gene pyramiding) for the development of bean germplasm or cultivars with enhanced resistance or combined resistance to several pathogens [6]. However, the efficiency of MAS for breeding disease resistance in common bean is still hampered due to the dominance of many RAPD/SCAR markers, repulsion phase linkage between markers and dominant traits, continued need for progeny testing, and the realization that some markers are loosely linked in distinct mapping populations and/or ineffective in specific market classes or gene pools [7, 9, 11, 14–16]. The development of novel disease resistance markers or the refinement of existing ones would benefit from the availability of highly polymorphic marker systems that generate codominant, more tightly linked, and broadly applicable markers.

Next-generation sequencing (NGS) allows the rapid development and application of genomics tools in plant breeding [17, 18]. It enables the inexpensive and rapid discovery of DNA polymorphisms such as simple sequence repeats (SSR), insertion-deletions (InDel), and single nucleotide polymorphisms (SNP) in model crops [19, 20], including common bean [21, 22]. Using the recently released reference genome sequence of common bean [23] markers can now be assigned physical genomic positions and candidate genes identified.

BSA and the NGS technology were recently applied to mapping and marker development for disease resistance loci in several crops [24–27]. By using restriction-site associated DNA (RAD) sequencing in a RIL population of lupin, markers tightly linked (0.5-0.9 cM) to anthracnose (*Colletotrichum lupini*) and phomopsis stem blight (*Phomopsis leptostromiformis*) resistances were rapidly developed [26, 27]. In rice, a major QTL for partial resistance to rice blast (*Magnaporthe oryzae*) was identified in a RIL population subjected to whole-genome sequencing [25]. In order to minimize costs for NGS sequencing of complex genomes, BSA has also been coupled with transcriptional profiles from RNA-Seq data for mapping of QTL and mutant genes of agronomic interest in crops [28–30].

As a result of massive discovery of polymorphisms by NGS, high-throughput genotyping platforms for genome-wide screening of SNPs were developed for crop species [20]. For common bean, a Golden Gate SNP assay containing 1,050 working SNPs, discovered by comparing SNPs between breeding lines Jalo EPP558 (Andean origin) and BAT93 (Middle American origin) was released [21]. Recently, the Common Bean Coordinated Agricultural Project (BeanCAP; http://www.beancap.org) consortium developed an Illumina Infinium BeadChip (BARCBEAN6K_3) containing 5,398 SNPs [31]. The SNPs were identified using whole-genome sequence analysis of 17 cultivars from different market classes and gene pools. This high-density SNP array provides greater whole genome coverage over previous marker systems. The BeanCAP diversity panel, consisting of over 500 common bean lines, was genotyped with the BARCBEAN6K_3 SNP chip and is being mainly utilized for association mapping studies and mapping select traits in bi-parental RIL populations [31–33].

The genome-wide SNP coverage of common bean provides an opportunity to discover SNPs underlying genomic regions of disease resistance traits previously identified by QTL mapping. A rapid method to discover or refine markers linked to common bean disease resistance genes would be the application of “*in silico* BSA”, which consists of inspecting SNP variation among genotyped individuals, with known phenotype, at specific targeted genomic regions. This approach circumvents the need to develop mapping populations or genotype the original population from which the gene or QTL region was identified.

Resistance to BCMV and *Bean common mosaic necrosis virus* (BCMNV) is genetically well defined, and conditioned by a single dominant (*I*) and four recessive (*bc-u, bc-1, bc-2, bc-3*) genes [34]. Different alleles were identified for two of the loci (*bc-1*^*2*^ and *bc-2*^*2*^). The chromosomal position of the *I* gene, *bc-1*^*2*^ and *bc-3* genes map independently [6]. The *I* gene confers immune or hypersensitive resistance to all strains of BCMV and BCMNV and is located on chromosome Pv02 [6] within a complex locus containing a cluster of nucleotide binding site-leucine rich repeat (NBS-LRR)-type disease resistance genes (R-genes) [35]. A SCAR marker (SW13) linked to the *I* gene was developed [36] and is widely used by bean breeders [6]. However, in several mapping populations, recombination between the SW13 marker and *I* gene was detected [15]. Furthermore, due to the dominant nature of the marker there is still the need to perform progeny testing in order to identify homozygous resistant plants. Therefore, a codominant marker more tightly linked to the *I* gene will be more informative than current dominant markers, and more accurate in predicting the disease reaction to BCMV.

As a proof of concept, *in silico* BSA was conducted on the BeanCAP panel to discover SNPs tightly linked to the chromosomal region underlying the *I* gene, with the aim of developing codominant markers with broad applicability for MAS of the *I* gene across the different market classes and gene pools of common bean. We discovered such SNPs and validated their linkage with the *I* gene segregating in a RIL population. Finally, KASP (Kompetitive Allele Specific PCR) and CAPS (Cleaved Amplified Polymorphic Sequence) markers were developed from one of the SNP markers tightly linked with the *I* gene and successfully validated in a subset of lines from the BeanCAP panel and in the RIL population.

## Results

### Identification of candidate disease-resistance genes at the *I*gene

The *I* gene, as determined by the sequence of PCR products from primers for *Phgp* and Bng45 [35], is a locus located in the distal arm of chromosome Pv02 which encompasses a genomic region of ~154 kb, between positions 48,131,655 and 48,285,850. This locus contains seven putative disease-resistance loci belonging to the NBS-LRR gene family (Phvul.002G323000 to Phvul.002G323500, and Phvul.002G323800) which spans a ~81.7 kb region (48,183,168- 48,264,877).

### Identification of SNP markers associated to BCMV resistance by *in silico*BSA

**Figure 1:**
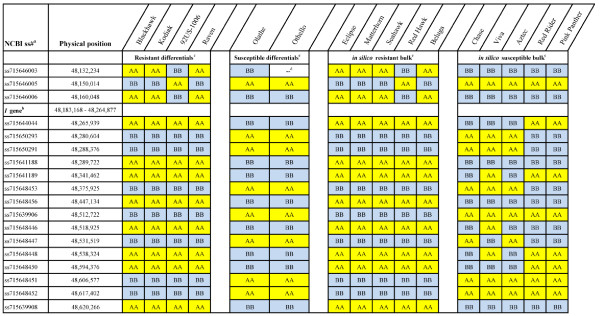
***In silico*** **resistant and susceptible bulks, and graphical genotypes of individual lines based on SNPs identified at the** ***I*** **gene.** ^a^NCBI Assay ID (ss#) at dbSNP Short Genetics Variation database (http://www.ncbi.nlm.nih.gov/projects/SNP/) containing the target SNP and flanking sequence information. ^b^The region contains seven putative disease-resistance loci belonging to the NBS-LRR gene family (Phvul.002G323000 to Phvul.002G323500, and Phvul.002G323800) which spans a ~81.7 kb region (48,183,168- 48,264,877) in chromosome Pv02. ^c^Cultivars for differential host reaction with the NL-3 strain of BCMNV [40]. ^d^Genotype allele call not determined. ^e^Composition of *in silico* resistant and susceptible bulks with cultivars of known disease reaction.

Although, no SNPs were found in either the coding or intergenic regions of the NBS-LRR loci, 18 SNPs were found in the BeadChip that were in close proximity to the cluster of NBS-LRR R-genes at the *I* gene (Figure 1). The closest and farthest SNPs mapped within ~51 and ~355 kb from proximal (Phvul.002G323000) and distal (Phvul.002G323800) R-genes, respectively.

Initially, when the two *in silico* bulks were screened for contrasting alleles, most resistant genotypes, with a few exceptions, exhibited a homogenous haplotype. In contrast, susceptible genotypes exhibited multiple haplotypes (Figure 1).

Only six SNPs were consistently associated with the *I* gene (Table 1). Further inspection for the remaining inoculated genotypes with known reaction to BCMV in the BeanCAP panel of 115 accessions (Additional file 1: Table S1) confirmed the association of the six SNPs to the phenotypic reaction to BCMV. Furthermore, the cosegregation of such SNPs with the *I* gene were validated *in silico* on the entire BeanCAP panel and the RIL lines genotyped on the BARCBEAN6K_3 chip (Additional file 1: Table S1 and Additional file 2: Table S2). The contrasting SNPs define two distinct haplotypes, each associated with resistance or susceptibility.

**Table 1:** **Summary of tightly linked SNP markers to the** ***I*** **gene associated to BCMV resistance**

BARCBEAN6K_3 SNP ID ^a^	SNP (NCBI ss#) ^b^	Susceptible SNP allele	Resistant SNP allele	Physical position	Distance from proximal and distal NBS-LRR R-genes ***I***locus ^c^
sc01349ln84482_14096_G_A_345640749	ss715641188	G	A	48,289,722	106,554/24,845
sc00445ln245016_77514_C_T_217829459	ss715648456	C	T	48,447,134	263,966/182,257
sc00445ln245016_132522_T_G_217884467	ss715639906	T	G	48,512,722	329,554/247,845
sc00445ln245016_228076_A_G_217980021	ss715648451	A	G	48,606,577	423,409/341,700
sc00445ln245016_240603_A_G_217992548	ss715648452	A	G	48,617,402	434,234/352,525
sc00445ln245016_243467_G_A_217995412	ss715639908	G	A	48,620,266	437,098/355,389

^a^Original SNP name in the BARCBEAN6K_3 SNP BeadChip.

^b^NCBI Assay ID (ss#) at dbSNP Short Genetics Variation database (http://www.ncbi.nlm.nih.gov/projects/SNP/) containing the target SNP and flanking sequence information.

^c^The region contains seven putative disease-resistance loci belonging to the NBS-LRR gene family (Phvul.002G323000 to Phvul.002G323500, and Phvul.002G323800) which spans a ~81.7 kb region (48,183,168- 48,264,877) in chromosome Pv02.

### Greenhouse screening for BCMV resistance

Greenhouse screening for BCMV/BCMNV resistance with strain NL-3 of BCMNV accurately discriminated between resistant genotypes [carrying the dominant allele (*I-*)] and susceptible (*ii*) genotypes. Systemic necrosis in genotypes with the *I* gene *(I-*) developed as early as five days post inoculation (dpi), whereas vein necrosis (*II bc-1*^*2*^*bc-1*^*2*^) and local lesions (*II bc-ubc-ubc-2*^*2*^*bc-2*^*2*^) developed within 10 dpi.

All BCMV differentials and genotypes from the BeanCAP panel initially screened in the greenhouse reacted as expected (Tables 2 and 3). In addition, the remaining BeanCAP genotypes, with known or unknown reaction to NL-3 strain, showed distinct disease symptoms that correlated with a haplotype associated with *I* gene resistance or susceptibility. All genotypes in this study appeared to be fixed for resistance (*II*) or susceptibility (*ii*), as all inoculated plants for each genotype displayed the same disease reaction.

**Table 2:** **Common bean genotypes of the BeanCAP panel, from Middle American origin, screened for BCMV resistance in the greenhouse and for markers linked to the** ***I*** **gene for resistance to BCMV**

Type	Genotype	Disease reaction ^a^	Genotype allele call for SNP BeadChip ^b^	SW13 ^c^	KASP ^d^	CAPS ^e^
Black	A-55	R	NA	+	A	A
	Black Magic	R	AA	+	A	A
	Blackhawk	R	AA	+	A	A
	Eclipse	R	AA	+	A	A
	Jaguar	R	AA	+	A	A
	Phantom	R	NA	+	A	A
	Raven	NR	AA	+	A	A
	UI-906	R	AA	+	A	A
	Zorro	R	AA	+	A	A
Black mottle	Orca	R	AA	+	A	A
Great Northern	ABC-Weihing	R	AA	-	A	A
	Beryl R	R	AA	+	A	A
	GN9-4	R	AA	+	A	A
	JM-24	R	AA	+	A	A
	Matterhorn	R	AA	+	A	A
	Orion	R	AA	+	A	A
Navy	Avalanche	R	AA	+	A	A
	Avanti	R	AA	+	A	A
	Huron	R	NA	+	A	A
	Newport	R	AA	+	A	A
	Seahawk	R	AA	+	A	A
Pink	USWA-61	R	AA	+	A	A
Pinto	Baja	R	AA	+	A	A
	Buster	R	AA	-	A	A
	JM-126	R	AA	+	A	A
	Kodiak	R	AA	+	A	A
	Quincy	R	AA	+	A	A
	Santa Fe	R	AA	+	A	A
	USPT-ANT-1	R	NA	-	A	A
	USPT-CBB-1	R	AA	-	A	A
	USPT-WM-1	R	AA	+	A	A
Small red	DOR 364	R	AA	-	A	A
	Rojo Chiquito	R	AA	+	A	A
Great Northern	ABCP-8	S	BB	-	G	B
	BelNeb-RR-1	S	BB	-	G	B
	BelNeb-RR-2	S	BB	-	G	B
	Chase	S	BB	-	G	B
	Emerson	S	NA	-	G	B
	GN Harris	S	BB	-	G	B
	GN#1Sel27	S	BB	-	G	B
	Sapphire	S	BB	-	G	B
	Starlight	S	BB	-	G	B
Pink	6R-42	S	BB	-	G	B
	Harold	S	BB	-	G	B
	ROG 312	S	BB	-	G	B
	Roza	S	BB	-	G	B
	Sutter Pink	S	NA	-	G	B
	UI-537	S	BB	-	G	B
	Viva	S	BB	-	G	B
	Yolano	S	BB	-	G	B
Pinto	AC Pintoba	S	BB	-	G	B
	Apache	S	BB	-	G	B
	Arapaho	S	BB	-	G	B
	Aztec	S	BB	-	G	B
	Bill Z	S	BB	-	G	B
	Burke	S	BB	-	G	B
	Common Pinto	S	BB	-	G	B
	Fiesta	S	BB	-	G	B
	Grand Mesa	S	BB	-	G	B
	Holberg	S	BB	-	G	B
	La Paz	S	BB	-	G	B
	Medicine Hat	S	BB	-	G	B
	Montrose	S	BB	-	G	B
	Nodak	S	BB	-	G	B
	NW-590	S	BB	-	G	B
	Othello	NR	BB	-	G	B
	Ouray	S	BB	-	G	B
	Pindak	S	NA	-	G	B
	Sierra	S	BB	-	G	B
	Topaz	S	BB	-	G	B
	UI-111	S	BB	-	G	B
	UI-114	S	BB	-	G	B
	UI-196	S	BB	-	G	B
Small Red	Garnet	S	BB	-	G	B
	USRM-20	S	BB	-	G	B

^a^R = Resistance (symptoms include systemic necrosis, mainly; and vein necrosis and local lesions, if *I* gene was protected by *bc* recessive genes); S = Susceptibility (systemic mosaic, or mild mosaic in the presence of *bc-1* ^*2*^ gene). NR = no disease reaction to the NL-3 strain of BCMNV due to the interaction of *I* allele and *bc-3* gene (R) or *bc-2* ^*2*^ gene alone (S).

^b^Automated genotype calls obtained from data of BARCBEAN6K_3 SNP BeadChip. NA = genotype not available. Sutter Pink is not part of the BeanCAP panel.

^c^Marker present = +; marker absent = -.

^d^KASP genotype calls. A = homozygous for SNP allele A; G = homozygous for SNP allele G.

^e^The CAPS marker yields two alleles. The resistant allele has two bands (201 and 110 bp), whereas the susceptible allele has a single band (311 bp). A = homozygous for resistant allele *II*; B = homozygous for susceptible allele *ii*.

**Table 3:** **Common bean genotypes of the BeanCAP panel, from Andean origin, screened for BCMV resistance in the greenhouse and for markers linked to the** ***I*** **gene for resistance to BCMV**

Type	Genotype	Disease reaction ^a^	Genotype allele call for SNP BeadChip ^b^	SW13 ^c^	KASP ^d^	CAPS ^e^
Cranberry	Capri	R	AA	+	A	A
	Cardinal	R	AA	+	A	A
	Dolly	R	AA	+	A	A
	Etna	R	AA	+	A	A
	Krimson	R	AA	+	A	A
	UI-51	R	AA	+	A	A
Dark Red Kidney	Fiero	R	AA	+	?	A
	Isles	R	AA	+	A	A
	Majesty	R	AA	+	A/G	A
	Montcalm	R	AA	+	A	A
	Red Hawk	R	AA	+	A	A
	Royal Red	R	AA	+	A	A
	USDK-CBB-15	R	AA	+	A	A
Light Red Kidney	Badillo	R	AA	+	A	A
	Blush	R	AA	+	A	A
	CELRK	R	AA	+	A	A
	Chinook 2000	R	AA	+	A	A
	Drake	R	AA	+	A	A
	K-42	R	AA	+	A	A
	Cardinal	R	AA	+	A	A
	Pink Panther	R	AA	+	A	A
	Red Kanner	R	AA	+	A	A
	Red Kloud	R	AA	+	A	A
White kidney	Beluga	R	AA	+	A	A
	Silver Cloud	R	AA	+	A	A
	USWK-CBB-17	R	AA	+	A	A
Cranberry	Cran-09	S	BB	-	G	B
	G122	S	BB	-	G	B
	G19833	S	NA	-	G	B
	Red Rider	S	BB	-	G	B
	Taylor Hort	S	BB	-	G	B
Dark Red Kidney	CDRK	S	BB	-	G	B
	Charlevoix	S	BB	-	G	B
Light Red Kidney	Sacramento	S	BB	+	G	B
	UC Red Kidney	S	BB	-	G	B
Pink cranberry	Ind. Jamaica Red	S	BB	-	G	B
Purple speckled	Jesca	S	NA	-	G	B
	KIJIVU	S	NA	-	G	B
Red	CANADA	S	NA	-	G	B
Red mottle	CAL-143	S	BB	-	G	B
	ICA Quimbaya	S	BB	-	G	B
	Pompadour B	S	BB	-	G	B
	ROZI KOKO	S	NA	-	G	B
White Kidney	Lassen	S	BB	-	G	B
Yellow	BUKOBA	S	NA	-	G	B
	Jalo EEP558	S	BB	-	G	B
	Myasi	S	BB	-	G	B

^a^R = Resistance (symptoms include systemic necrosis, mainly; and vein necrosis and local lesions, if *I* gene was protected by *bc* recessive genes); S = Susceptibility (systemic mosaic, or mild mosaic in the presence of *bc-1* ^*2*^ gene). NR = no disease reaction to the NL-3 strain of BCMNV due to the interaction of *I* allele and *bc-3* gene (R) or *bc-2* ^*2*^ gene alone (S).

^b^Automated genotype calls obtained from data of BARCBEAN6K_3 SNP BeadChip. NA = genotype not available. G19833 (used as reference for whole-genome sequence of common bean) is not part of the BeanCAP panel. In addition, African lines: Jesca, KIJIVU, CANADA, ROZI KOKO, and BUKOBA, belong to the Andean Diversity Panel of common bean.

^c^Marker present = +; marker absent = -.

^d^KASP genotype calls. A = homozygous for SNP allele A; G = homozygous for SNP allele G; ? = failed genotyping reaction. A/G = heterozygous.

^e^The CAPS marker yields two alleles. The resistant allele has two bands (201 and 110 bp), whereas the susceptible allele has a single band (311 bp). A = homozygous for resistant allele *II*; B = homozygous for susceptible allele *ii*.

### Conversion of select SNP into KASP and CAPS markers

The closest SNP (A/G) that consistently cosegregated with the *I* gene was ss715641188 (~24.85-31.6 kb from 5′ and 3′ ends of Phvul.002G323800, respectively), and therefore it was targeted for development of KASP and CAPS markers. The G allele was associated with susceptible genotypes, whereas the resistant genotypes were associated with A allele. The reference genotype G19833, used for whole genome sequencing of *P. vulgaris*, has the G allele. A KASP marker assay was developed, and select lines with known disease reaction to BCMV were initially screened. This allelic-specific assay detected each allele cosegregating with either *I* gene resistance (*II*) or susceptibility (*ii*) in all genotypes screened with the KASP marker (Tables 2, 3, and 4; Additional file 2: Table S2).

**Table 4:** **Summary of markers linked to the** ***I*** **gene for resistance to BCMV screened on the RIL population derived from G122 × Montcalm**

Genotype ^a^	Number of lines ^b^	Genotype allele call for SNP BeadChip ^c^	SW13 ^d^	KASP ^e^	CAPS ^f^
Montcalm (R)		AA	+	A	A
G122 (S)		BB	-	G	B
Resistant RILs	63	All AA	All +	All A	All A
Susceptible RILs	27	All BB	1 +/26 -	All G	All B

^a^R = Resistance (symptoms include systemic necrosis, mainly due to unprotected *I* gene); S = Susceptibility (systemic mosaic from lack of *I* gene or any recessive resistance genes).

^b^Number of lines screened with markers SW13, KASP, and CAPS in this study.

^c^Automated genotype calls obtained from data of BARCBEAN6K_3 SNP BeadChip.

^d^Marker present = +; marker absent = -.

^e^KASP genotype calls. A = homozygous for SNP allele A; G = homozygous for SNP allele G.

^f^The CAPS marker yields two alleles. The resistant allele has two bands (201 and 110 bp), whereas the susceptible allele has a single band (311 bp). A = homozygous for resistant allele *II*; B = homozygous for susceptible allele *ii*.

**Figure 2:**
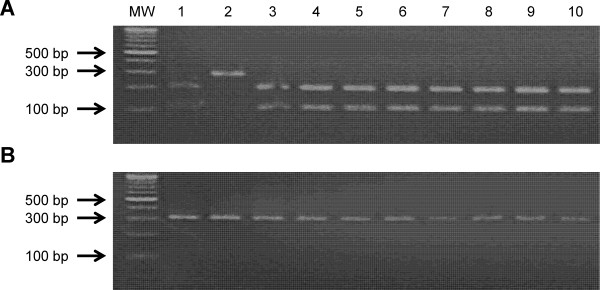
**Restriction digestion pattern for allele-specific CAPS marker targeting SNP ss715641188 linked to** ***I*** **gene controlling resistance to BCMV.** MW =100 bp DNA ladder. Digestion of PCR amplicon (311 bp) with *Taq*I generates products of 201 and 110 bp. **(A)** 1. BCMV-resistant line Montcalm (*II*), 2. BCMV-susceptible line G122 (*ii*), 3-10. Resistant RILs. **(B)**. 1-10. Susceptible RILs.

Amplification with the primers developed for the CAPS marker produced a single PCR amplicon of the expected 311 bp size. Digestion of the PCR products for select lines with *Taq*I endonuclease detected the SNP associated with the BCMV-resistant allele (*II*), generating two bands of 201 and 110 bp. In contrast, PCR products from susceptible lines (*ii*) were not cut by *Taq*I due to the alteration of the restriction site (Figure 2). As expected, this marker was able to distinguish between resistant and susceptible genotypes in both the BeanCAP panel and the RIL population (Tables 2, 3, and 4; Additional file 2: Table S2).

### Linkage between marker and *I*gene locus

Initially, we screened the BeanCAP genotypes and RIL population with SW13 marker, which is a dominant marker in coupling phase with the *I* gene. In general, the presence or absence of SW13 marker corresponded to the known phenotype for plants screened in the greenhouse (Tables 2 and 3). However, the SW13 marker was not detected in five resistant genotypes from the subset of the BeanCAP collection, belonging to races Durango/Jalisco of the Middle American gene pool; also, the SW13 marker was present in a single susceptible RIL individual (GM-23; Additional file 2: Table S2) .

All 122 genotypes screened in the greenhouse for BCMV resistance (Tables 2 and 3), and 90 RILs from the G122 × Montcalm population, were also genotyped with the KASP and CAPS markers. The KASP assay distinguished resistant and susceptible genotypes in all cases. Likewise, the CAPS marker completely distinguished resistant (*II*) or susceptible (*ii*) genotypes. Finally, based on the cosegregation of phenotype and SNP markers in the RIL population, the SNP was tightly linked to the genomic region underlying the *I* gene as no recombinant individuals were detected.

## Discussion

We used *in silico* BSA to discover SNP variation within the genomic region associated with BCMV resistance in a diverse collection of germplasm. This approach led to the development of a tightly linked-codominant genetic marker. Our results demonstrate that we can take advantage of the extensive information of gene/QTL mapping for disease resistance traits in common bean [5, 6] and the power of *in silico* BSA of NGS data as a rapid and low-cost approach for marker development.

The rate of marker discovery in common bean was hampered in the past by a lack of a reference genome sequence [23]. Now, a high-density SNP array platform facilitates high-throughput genotyping. Multiple diversity panels containing hundreds of lines from diverse breeding programs have been densely genotyped with thousands of markers and can now be applied to genetic studies for which multiple sets of phenotypic data can be collected. Here the availability of these genomics resources allowed us to map the physical position of the *I* gene. The region underlying the *I* gene contains a cluster of seven NBS-LRR R-genes, spanning ~81.7 kb region and confirms previous findings [35] that suggested that this region was enriched for NBS-LRR-type sequences.

BSA, coupled with NGS genotyping pipelines, has been applied in other crops for development of markers linked to disease resistance genes [24–27] using contrasting (i.e., resistant vs. susceptible) pools of RIL populations. In several instances, specific RIL populations segregate for only one or few genetic traits. In contrast, we applied *in silico* BSA to the BeanCAP panel, containing 506 lines genotyped on an Infinium 6K SNP BeadChip, which circumvented the need to develop a bi-parental mapping population. In addition, the use of the diversity panel allowed us to explore many more recombination events. Most SCAR markers linked to disease resistance traits in common bean (http://www.ars.usda.gov/SP2UserFiles/person/3848/PDF/Miklas_2011/SCAR%20Markers%202010.pdf) were developed by BSA using RAPD markers and RIL populations, many of them derived from wide crosses [5, 6]. Consequently, several markers were of use only in specific populations, market classes, or gene pools (e.g., SCAR marker SAS8, for *Beet Curly Top* virus resistance, works only in germplasm from Andean origin) [5, 6, 14]. The SNPs, and subsequent KASP and CAPS markers cosegregated with the *I* gene in the full BeanCAP panel and a RIL population (Additional file 1: Table S1 and Additional file 2: Table S2).

Although the SW13 marker is widely used in common bean breeding programs and seems to work across diverse material, recombination between the marker and *I* gene has been detected in distinct mapping populations, ranging from 1.3 to 8.9 cM [15, 36, 37]. In fact, five resistant members of the diversity panel were assigned as susceptible and a single susceptible RIL line was erroneously scored as resistant, based on the absence or presence of the dominant SW13 marker, respectively. The latter event is of greater consequence during breeding since a truly susceptible line might be advanced in the program even though it otherwise had the unwanted genotype (*ii*). The new markers described here resolve those issues for breeders.

A BLAT search on the reference genome database revealed that the SW13 marker was distributed along several common bean chromosomes. The closest sequence hits [Expect (E)-value 1.00^−192^, identity 94.4% (357/358 bp), and E-value 1.00^−103^, identity 94.3% (200/212 bp)] for partial sequences of SW13 marker on chromosome Pv02 are approximately between 6 and 12 Mbp away from the *I* gene. The position of the ss715641188 SNP to the closest R-gene model (Phvul.002G323800) is ~25 kb, which suggests the chance for recombination between the SNP marker and the most proximal R-gene is very minimal.

We have demonstrated that BSA coupled with NGS technology can accurately identify SNPs linked to a disease resistance trait, which in turn enabled the development of robust marker systems. The approach to tag monogenic and quantitative traits of agronomic interest in common bean previously relied on the application of BSA using AFLP, RAPD, or SSR markers, but this task proved to be tedious and time consuming. The main advantage of NGS data is that sequence surrounding SNPs or any sequenced marker is readily available and can be assigned to a physical position on the reference genome; thus, their conversion to effective genetic markers is relatively straight forward.

We decided to target the closest candidate SNP (ss715641188), that consistently cosegregated with *I* gene, for conversion to a cost-effective genetic KASP and CAPS markers. Both genotyping technologies accurately predicted the disease response phenotype. In addition, these markers are codominant and thus are able to distinguish between heterozygous and homozygous resistant plants. Thus, the implementation of either KASP or CAPS screening procedure can select homozygous resistant plants during early breeding cycles (e.g., F_2_ selection). This will save at least four months of progeny testing for this phenotype [15].

BCMV is one of the most important viral diseases of common bean worldwide, and the combination of resistance genes with different mechanisms of action offers a more durable resistance compared to single gene resistance [38]. In addition, where necrotic strains of BCMV or BCMNV are present, plants with the *I* gene need to be protected from the strong, hypersensitive response by the addition of *bc* recessive genes [8]. One of the most desirable gene combinations is that of *I* + *bc-3*. However, the action of the *I* gene is masked by *bc-3*, and therefore direct selection for *I* gene is not possible [38]. Thus, these new markers offer an opportunity for more efficient indirect selection of the *I* gene in the presence of *bc* recessive genes and greatly facilitates pyramiding the appropriate combination of genes.

## Conclusions

This is the first BSA approach that makes use of the wealth of information of gene/QTL mapping for disease resistance traits in common bean and exploits the SNP variation underlying the chromosomal region associated with the *I* gene in a diversity panel. Many disease resistance-markers previously developed for common bean are not ideal for MAS (e.g., dominant, loose linkage, market class-specific). In this study, we were able to identify two distinct haplotypes each associated to resistance or susceptibility to BCMV across genetically diverse germplasm. Since the physical position and sequences flanking the informative SNPs are known, the task for development of robust codominant marker systems is simplified. In addition, the availability of a densely phenotyped/genotyped diversity panel guarantees that developed markers are applicable to diverse germplasm, thus circumventing the need for developing specific bi-parental mapping populations.

## Methods

### Plant materials and DNA isolation

A total of 122 diverse genotypes of common bean, including 115 lines of the BeanCAP and five African lines of the Andean Diversity Panel (ADP; http://arsftfbean.uprm.edu/bean/?page_id=179), from different market classes, belonging to both Middle American (black, navy, small white, Great northern, pink, pinto, small red) and Andean (cranberry, dark red kidney, light red kidney, white kidney, red, red mottle, purple speckled, yellow) gene pools, with known or unknown disease reaction to BCMV were evaluated in this study (Tables 2 and 3; Additional file 1: Table S1). The universal BCMV-susceptible cultivar Sutter Pink, and the line G19833 (which was used as reference for whole-genome sequence of *P. vulgaris*) were utilized in this study. In addition, an advanced F_2:5_ derived RIL [GM; G122 (susceptible) × Montcalm (resistant)] population of 98 lines, and the parental lines, were also included in this study for validation of marker-*I* gene cosegregation (Additional file 2: Table S2). The GM population shows distorted segregation in favor of the *I* gene, which is a common occurrence previously documented in distinct RIL populations [32, 39].

Genomic DNA was extracted from the first trifoliate leaves of two-week old plants using the DNeasy Plant Mini Kit (Qiagen, Valencia, CA) according to the manufacturer’s protocol. For marker validation, DNA was extracted from pooled-trifoliate leaves of up to four plants per genotype before viral inoculation procedure.

### Plant genotyping and *in silico*BSA

Five-hundred and six genotypes that compose the BeanCAP panel were previously genotyped with the Illumina BARCBEAN6K_3 Infinium SNP BeadChip, which includes allelic data for 5,398 SNP. Genotyping was performed according to manufacturer’s recommendations at the USDA Soybean Genomics and Improvement Lab, Beltsville, MD, and data generated for each genotype was used for allele calling using GenomeStudio Software v2011.1 (Illumina, San Diego, CA).

The physical position for all SNPs in the BeadChip in the whole genome sequence of common bean (http://www.phytozome.net/commonbean) is known. The genomic region for the *I* gene was identified by querying the sequences of markers *Phgp* and Bng45 [35], flanking the region associated to BCMV resistance, against the *P. vulgaris* reference genome database in Phytozome using BLAT search.

BCMV differentials [i.e., Blackhawk (*II*), Kodiak (*II bc-1*^*2*^*bc-1*^*2*^), 92US-1006 (*II bc-2*^*2*^*bc-2*^*2*^), Raven (*II bc-3bc-3*), Olathe (*ii bc-1*^*2*^*bc-1*^*2*^), and Othello (*ii bc-2*^*2*^*bc-2*^*2*^) [40] and ten BeanCAP genotypes with known disease reactions to strain NL-3 of BCMNV were divided into resistant and susceptible *in silico* bulks. Resistant genotypes (dominant *II* gene) included were: Eclipse, Matterhorn, Seahawk, Red Hawk, and Beluga; whereas susceptible genotypes (recessive *ii* gene) included were: Chase, Viva, Aztec, Red Rider, and Pink Panther. Then, each bulk was inspected for SNP variation at the *I* gene and flanking regions. Contrasting SNP alleles between the *in silico* bulks were considered as putative markers linked to BCMV resistance. After the initial *in silico* BSA, the rest of the BeanCAP panel, including the RIL population, was inspected for haplotypes defining the resistant and susceptible groups (Additional file 1: Table S1 and Additional file 2: Table S2).

### BCMV resistance screening

Seventy five and 47 genotypes from both Middle American and Andean origin, respectively, with either the resistant- or susceptible-haplotype were screened in the greenhouse for *I* gene resistance with NL-3 strain of BCMNV. The Andean G19833 line and Middle American Sutter Pink cultivar were also screened. The RIL population was previously screened for BCMV resistance (*unpublished data*).

BCMNV NL-3 strain was maintained in infected seeds of Sutter Pink, and young leaflets of plants showing mosaic symptoms were used as inoculum. The disease reaction of each genotype was verified by mechanical inoculation with the virus [34, 38], and cultivars for BCMV differential genotypes were used as controls [40]. Homozygous recessive (*ii*) plants without recessive genes develop systemic mosaic symptoms, but those homozygous plants carrying the *bc-u bc-1*^*2*^ or *bc-u bc-2*^*2*^ develop mild mosaic or no symptoms, respectively. In contrast, plants carrying a dominant allele (*I-*) develop systemic necrosis and eventually die, and depending upon the presence of other recessive *bc-*genes plants develop vein necrosis (*II bc-1*^*2*^*bc-1*^*2*^) or local lesions (*II bc-u bc-u bc-2*^*2*^*bc-2*^*2*^). However, plants with the *I* gene in the homozygous background of *bc-3* gene develop no symptoms [38].

In short, the primary leaves of four 10-day seedlings of each genotype, growing in a 10 × 10 cm plastic pot containing Sunshine Professional Growing Mix (Sun Gro Horticulture, Bellevue, WA) were rub inoculated with the viral inoculum, and incubated in the greenhouse (24-28°C, 14-h photoperiod). The disease reaction was periodically assessed five dpi until 4-weeks dpi, and each genotype was classified as susceptible or resistant according to the symptoms described above.

### Development and validation of genetic KASP and CAPS markers

Only the closest SNP marker [ss715641188 (sc01349ln84482_14096_G_A_345640749)] to the *I* gene was targeted for conversion to KASP (LGC Genomics, Beverly, MA) and CAPS markers. Allele-specific reverse KASP primers (BCMV_48289723_A, BCMV_48289723_G), and a common forward primer (BCMV_48289723_F) were designed (Ag-Biotech, San Juan Bautista, CA) (Table 5) and initially validated in the genotyped BCMV differentials, G19833, and the RIL parental lines. The KASP genotyping reaction and PCR thermocycling conditions were carried out according to manufacturer’s recommendations (LGC Genomics, Beverly, MA). PCR reaction mixture was carried out in a 10 μl-volume consisting of 2.5 μl DNA (10 ng μl^−1^), 2.5 μl 2X KASP Master mix v4.0, and 0.07 μl 72X KASP Primer mix. PCR amplification and fluorescent end-point genotyping was carried out in a LightCycler 480 thermocycler (Roche Applied Science, Indianapolis, IN). Primer sequences were used as queries in a BLAST search to check primer specificity against the common bean genome sequence database.

**Table 5:** **Sequences of primers used for genotyping BCMV resistance conferred by the** ***I*** **gene**

Marker	Primers (5′-3′)	Physical position
SW13 marker	CACAGCGACATTAATTTTCCTTTC	Multiple hits in the genome
(Melotto et al., 1996) [36]	CACAGCGACAGGAGGAGCTTATTA	
KASP marker (without tail sequences),		
BCMV_48289723_F	CCCTAATTCACTTTCCGAGTAAGAGAAGC	48,289,632
BCMV_48289723_G	TGAAAATGGGTCGGGTCGGAC	48,289,742
BCMV_48289723_A	CTTGAAAATGGGTCGGGTCGGAT	48,289,744
CAPS marker		
BCMV_48289723_CAPS	AGGAGGAAGAACGGTGGTC	48,289,519
	TTTGGTGGTAATTTGAAAATGG	48,289,829

For CAPS marker (Table 5), the SNP was analyzed for potential restriction enzyme recognition sites using the SGN CAPS designer (http://sgn.cornell.edu/tools/caps_designer/caps_input.pl). Several restriction endonucleases were found to recognize the SNP (A/G) and neighboring bases, and *Taq*I was chosen for the CAPS genotyping assay as it is commonly used and low-cost. *Taq*I recognizes the restriction site TCGA and cleaves the resistance allele but not the susceptible allele due to the alteration of the restriction site (i.e., TCGG) by the SNP. A PCR primer pair (BCMV_48289723_CAPS) flanking the SNP was designed using Geneious R7 (Biomatters Inc., San Francisco, CA) to produce a PCR amplicon of 311 bp. Primer specificity was checked against the common bean genome sequence database. PCR reaction mix consisted of 1X Go Taq Reaction buffer, 1.5 mM MgCl_2_, 0.2 mM of each dNTP’s, 0.2 μM each primer, 20 ng genomic DNA, and 1 U of Go Taq DNA polymerase, in a 25 μl final volume. All reagents were purchased from Promega (Madison, WI). PCR was performed on an GeneAmp PCR System 9700 thermocycler (Applied Biosystems, Foster City, CA) with an initial denaturation at 94°C for 5 min; followed by 35 cycles of 94°C for 30s, 58°C for 30 s, and 72°C for 30 s; final step of 72°C for 5 min. Initially, PCR products were separated and visualized on a 1% agarose gel to verify presence and correct size of PCR amplicon. A five-μl aliquot of PCR products were digested in a 20 μl reaction containing 1X FastDigest Green buffer, and 1 μl FastDigest *Taq*I enzyme (Thermo Scientific, Waltham, MA). The reaction was incubated at 65°C for 15 min on the thermocycler, and digestion products were separated and visualized on a 2% agarose gel. The resistant allele is cleaved by *Taq*I generating two bands of 201 and 110 bp.

After initial validation of KASP and CAPS markers, all 122 genotypes (including those from the BeanCAP and ADP panels, Sutter Pink and G19833), RIL population (n = 92) and its parental lines were genotyped with the above markers. In addition, plants were also screened with the dominant SW13 SCAR marker, which generates a 690 bp PCR amplicon [36], using the same PCR thermocycling conditions as those for the CAPS marker, except that the extension time was at 72°C for 1 min. The recombination frequency between markers (SNP ss715641188, SW13) and the *I* gene in the RIL population was calculated by χ^2^ analysis.

## Electronic supplementary material

Additional file 1: Table S1: Graphical genotypes of lines screened in the greenhouse and all BeanCAP accessions determined by SNPs flanking the *I* gene. (XLSX 536 KB)

Additional file 2: Table S2: Graphical genotypes of RILs, derived from the cross of G122 × Montcalm, determined by SNPs flanking the *I* gene. (XLSX 25 KB)
